# Functional Biopolymer-Stabilized Silver Nanoparticles on Glassy Carbon: A Voltammetric Sensor for Trace Thallium(I) Detection

**DOI:** 10.3390/ijms26199658

**Published:** 2025-10-03

**Authors:** Bożena Karbowska, Maja Giera, Anna Modrzejewska-Sikorska, Emilia Konował

**Affiliations:** Faculty of Chemical Technology, Poznan University of Technology, Berdychowo 4, 60-965 Poznan, Poland; bozena.karbowska@put.poznan.pl (B.K.); anna.modrzejewska-sikorska@put.poznan.pl (A.M.-S.)

**Keywords:** differential pulse anodic stripping voltammetry, electrode modification, thallium, silver nanoparticles, potato starch derivatives, environmental samples

## Abstract

Thallium is a soft metal with a grey or silvery hue. It commonly occurs in two oxidation states in chemical compounds: Tl^+^ and Tl^3+^. Thermodynamically, Tl^+^ is significantly more stable and typically represents the dominant form of thallium in environmental systems. However, in this chemical form, thallium remains highly toxic. This study focuses on the modification of a glassy carbon electrode (GCE) with silver nanostructures stabilised by potato starch derivatives. The modified electrode (GCE/AgNPs-E1451) was used for the determination of trace amounts of thallium ions using anodic stripping voltammetry. Emphasis was placed on assessing the effect of surface modification on key electrochemical performance parameters of the electrode. Measurements were carried out in a base electrolyte (EDTA) and in a real soil sample collected from Bali. The stripping peak current of thallium exhibited linearity over the concentration range from 19 to 410 ppb (9.31 × 10^−8^ to 2.009 × 10^−6^ mol/dm^3^). The calculated limit of detection (LOD) was 18.8 ppb (9.21 × 10^−8^ mol/dm^3^), while the limit of quantification (LOQ), corresponded to 56.4 ppb (2.76 × 10^−7^ mol/dm^3^). The GCE/AgNPs-E1451 electrode demonstrates several significant advantages, including a wide detection range, reduced analysis time due to the elimination of time-consuming pre-concentration steps, and non-toxic operation compared to mercury-based electrodes.

## 1. Introduction

Thallium is a soft metal with a gray or silvery appearance. In chemical compounds, it typically occurs in two oxidation states. The monovalent form (Tl^+^), found in compounds such as Tl_2_O and Tl_2_SO_4_—or in Tl_2_S under strongly reducing conditions—is by far the more stable and prevalent form. The trivalent state (Tl^3+^), observed under oxidizing and alkaline conditions in compounds like Tl_2_O_3_, Tl(OH)_3_, and TlCl_3_, is much less common. From a thermodynamic perspective, Tl^+^ is significantly more stable and represents the dominant chemical form of thallium in nearly all environmental settings [1].

Until the early 20th century, thallium was widely used in medicine, particularly for treating scalp fungal infections, tuberculosis, malaria, and venereal diseases. Its application extended well into the 1960s, during which thallium salts were still used as rodenticides and insecticides [2]. However, in 2014, the European Cooperation in Science and Technology (COST TD1407) classified thallium as a technology-critical element—highlighting its environmental hazards and potential risks to both human and animal health.

Among heavy metals, thallium ranks as one of the most toxic—surpassing even mercury, cadmium, lead, copper, and zinc in terms of its harmful effects on the human body. This high toxicity is largely attributed to the chemical resemblance between the Tl^+^ ion and potassium: they share similar ionic radii and charge, which allows thallium to interfere with essential biological processes [3].

Thallium is a cumulative poison, capable of building up in living organisms over time. Exposure may occur through various routes: ingestion of contaminated food, dermal contact, inhalation of dust or fumes, or absorption through the respiratory tract. Once inside the body, the severity of poisoning depends on the dose, individual immune response, and personal sensitivity to the toxin. Symptoms of thallium poisoning are typically diverse and nonspecific, which complicates diagnosis and treatment [4].

For this reason, the detection and quantification of thallium ions is of critical importance. Despite its extreme toxicity, thallium tends to occur at relatively low concentrations in the environment, making its identification particularly challenging. Accurate detection, therefore, requires highly sensitive analytical techniques capable of determining trace amounts of metals and their compounds in environmental, clinical, or industrial samples.

Inductively coupled plasma mass spectrometry (ICP-MS) is currently considered one of the most advanced techniques for trace analysis. Flame atomic absorption spectrometry (FAAS) is also frequently used to determine thallium in environmental samples. Electrothermal atomic absorption spectrometry (ETAAS) is a well-established technique for monitoring trace amounts of elements in nearly all types of matrices. Additionally, Zeeman-effect electrothermal atomic absorption spectrometry (ZEETAAS) has been used to determine thallium in water samples, often in combination with flotation or other pre-concentration procedures before analysis [5,6,7].

However, electroanalytical methods, especially voltammetry, have proven particularly effective, simple and inexpensive in this regard.

Voltammetric techniques are based on the relationship between current and the concentration of electroactive species present in a solution. Among them, anodic stripping voltammetry (ASV) stands out for its high sensitivity. In ASV, the preconcentration step occurs both at the electrode surface and throughout its volume, which significantly enhances sensitivity and lowers detection limits—down to concentrations as low as 10^−12^ M in the case of environmentally relevant metals.

A crucial factor in successful voltammetric analysis is the appropriate selection of the working electrode. Over the past several decades, a variety of solid electrodes have been employed for trace metal detection. Commonly used types include electrodes made of noble metals (such as silver and gold), those based on transition metals (e.g., bismuth films or antimony-based electrodes), as well as carbon electrodes modified with electrolytically deposited mercury films (ex situ mercury film electrodes) [8,9,10,11].

Furthermore, electrode performance can be enhanced through surface modification—for instance, by applying thin layers of metallic nanostructures or other compounds [12,13,14].

As previously noted, thallium occurs in the environment in two oxidation states: monovalent (Tl(I)) and trivalent (Tl(III)), with the latter being approximately four times more toxic to humans and animals. Speciation of thallium remains challenging due to its presence at trace concentrations. Several reports have demonstrated that inductively coupled plasma mass spectrometry (ICP-MS) provides a reliable approach for the analysis of this metal. Szopa and Michalski [15] highlighted the key advantages of ICP-MS, including exceptionally low detection and quantification limits, minimal spectral interference, and high precision and reproducibility of measurements. Prior to analysis, Tl(I) and Tl(III) can be separated using cation-exchange shielding, ion-exchange resins, anion-exchange chromatography, or size-exclusion chromatography [16]. More recent studies have suggested that anionic surfactants, such as sodium dodecyl sulfate (SDS), may further facilitate the separation of thallium species during solid-phase extraction [17]. In addition, Choi et al. [18] reported the development of a Tl^3+^-selective probe synthesised from 2-acetyl-6-methoxynaphthalene and hydrazine. In an acetate buffer (pH 4.8) containing 1% (*v*/*v*) N,N-dimethylformamide as a solubiliser, this probe exhibited fluorescence signalling behaviour that was specifically activated in the presence of Tl^3+^.

This study focused on the determination of the total content of thallium cations. The applied sample mineralization procedure, with the addition of ascorbic acid after the process, led to the reduction of Tl^3+^ to Tl^+^. Consequently, the total thallium was determined in the form of Tl^+^. At the same time, a novel approach to electrode surface modification was introduced, based on silver nanostructures stabilized with a starch, aimed at enhancing the sensitivity of voltammetric detection of thallium ions.

## 2. Results

### 2.1. Selection of Deposition Potential for the Modified Electrode

**Figure 1:**
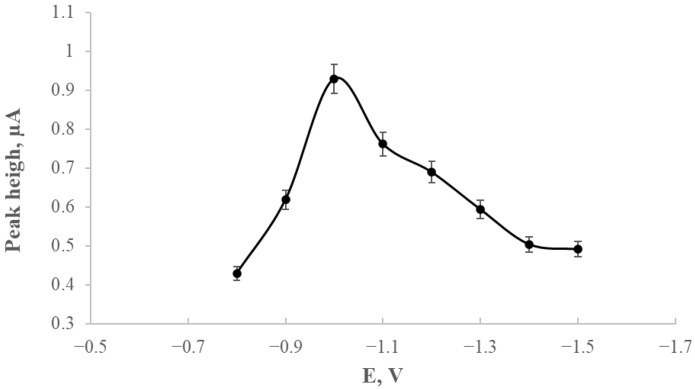
Thallium peak height as a function of the preconcentration potential in the base electrolyte (0.2 M EDTA) containing 200 µg/L of thallium. The preconcentration time was fixed at 120 s, while the deposition potential was varied between −0.8 V and −1.5 V versus SCE.

Following the electrode modification, experimental work was undertaken to determine the optimal deposition potential for thallium. The influence of deposition potential on the thallium peak height was evaluated relative to the saturated calomel electrode (SCE) over a potential range from −0.8 V to −1.5 V. The deposition time was fixed at 120 s, and the thallium concentration in the supporting electrolyte was maintained at 200 µg/L. Figure 1 presents the relationship between the thallium peak current and the applied deposition potential.

The highest thallium peak was obtained at a preconcentration potential of −1.0 V. Under these conditions, the thallium peak was best defined and increased proportionally with both the analyte concentration and the preconcentration time. Subsequent measurements were carried out at this potential of −1.0 V.

### 2.2. Dependence of Tl Peak Height on Deposition Time for the GCE/AgNPs-E1451 Electrode

**Figure 2:**
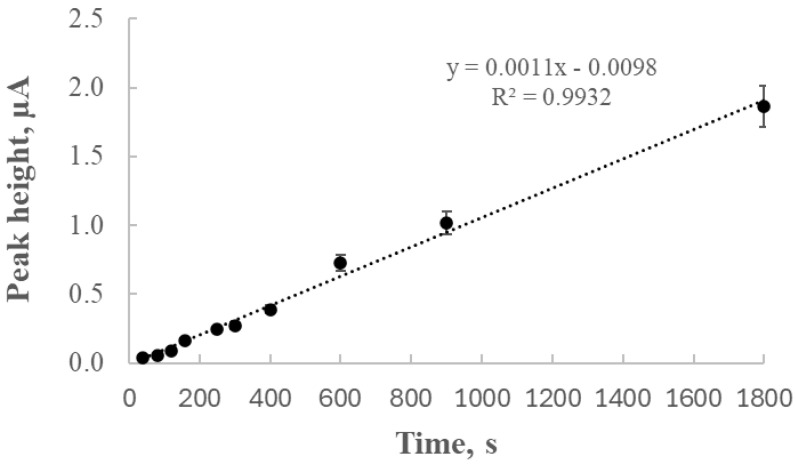
Relationship between thallium peak current and deposition time in a 0.2 M EDTA electrolyte containing 50 μg/L thallium. The deposition time ranged from 40 to 1800 s, with a deposition potential of −1.0 V. The measurements were performed using a step potential of 5 mV and a differential pulse amplitude of 25 mV.

The experiment was conducted at the previously determined preconcentration potential of −1.0 V. The thallium concentration in the tested system was 50 μg/L. The deposition time was varied from 40 s to 1800 s. Figure 2 shows the results graphically, presenting the relationship between the thallium peak height and the deposition time.

The linear relationship between the signal and deposition time observed from 40 to 1800 s indicates that thallium was not being depleted during this period. This correlation between the thallium peak height and deposition time allows for determining the optimal duration of preconcentration needed to obtain a peak with an easily measurable height.

Beyond this time range, the electrode material may have started to degrade, which was reflected by a decrease in signal intensity and a loss of linearity.

### 2.3. The Relationship Between Peak Current and Tl Concentration for the GCE/AgNPs-E1451 Electrode

**Figure 3:**
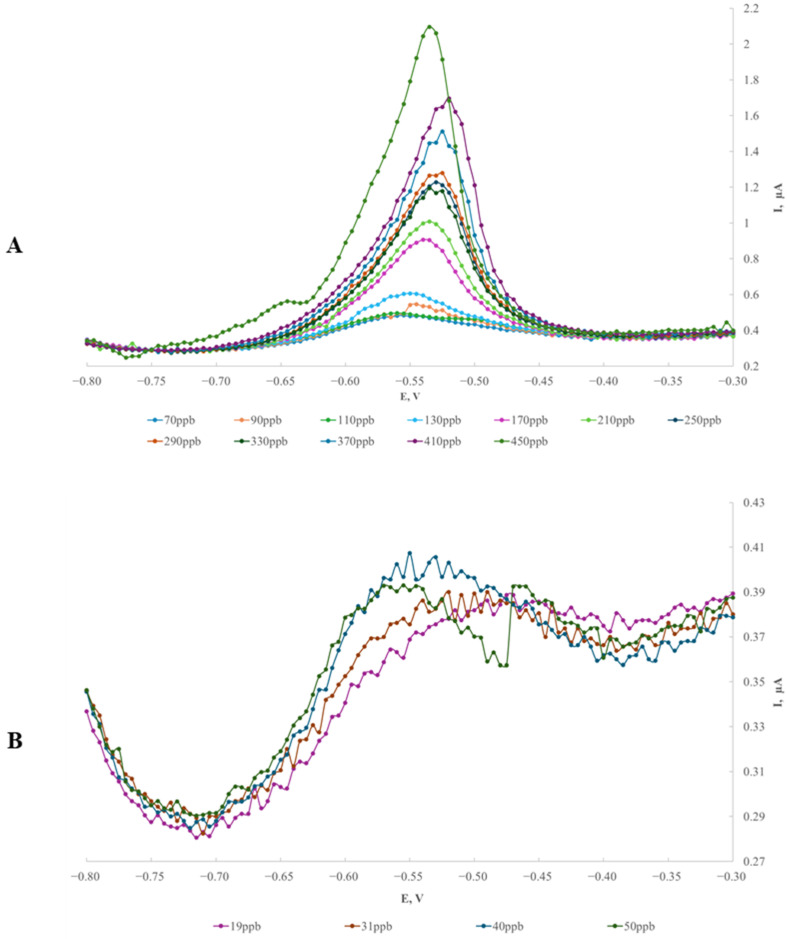
DPV spectra obtained using the GCE/AgNPs-E1451 modified electrode in 0.2 M EDTA solution. Thallium concentrations ranged: (**A**) from 70 ppb to 450 ppb, and (**B**) from 19 ppb to 50 ppb.

**Figure 4:**
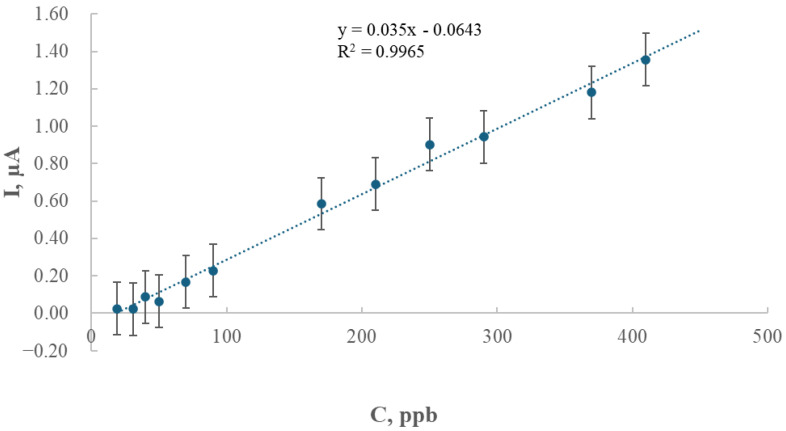
Plot of the peak current versus thallium concentration in 0.2 M EDTA electrolyte. The thallium concentration ranged from 19 μg/L to 410 μg/L, with a deposition time of 120 s and a deposition potential of −1.0 V. Measurements were performed using a step potential of 5 mV and a differential pulse amplitude of 25 mV.

A series of measurements was performed to establish the calibration curve. The thallium preconcentration time was set to 120 s, and the preconcentration potential was fixed at −1.0 V versus the calomel electrode. The resulting voltammograms for concentrations ranging from 19 μg/L to 410 and 450 μg/L are shown in Figure 3 and Figure 4, respectively.

During successive additions of thallium solution to 0.2 M EDTA, a clear response was recorded, demonstrating the excellent electroanalytical performance of the modified GCE/AgNPs-E1451 electrode. This is evidenced by the linear relationship between the current and increasing thallium concentration, tested over the range of 19 to 410 μg/L (ppb), corresponding to 9.31 × 10^−8^ to 2.009 × 10^−6^ mol/dm^3^ (Figure 4).

Using the calibration curve, the equation y = 0.035x − 0.0643 was obtained, with a correlation coefficient (R^2^) of 0.9965. The detection limit (LOD) was calculated based on the regression parameters of the calibration curve, according to the following formula:

LOD = (ĸ × SD_a_)/b,

where κ equals 3.3; SD_a_ is the standard deviation of the intercept, and b is the slope of the calibration curve. The calculated detection limit (LOD) based on this equation was 9.21 × 10^−8^ mol/dm^3^ (18.8 μg/L), while the limit of quantification (LOQ), defined as three times the LOD, corresponded to 2.76 × 10^−7^ mol/dm^3^ (56.4 μg/L).

The measurements were performed using differential pulse anodic stripping voltammetry (DP-ASV). The analyses were carried out in a nitrogen-deoxygenated 0.2 M EDTA solution at pH 4.5, which served as the base electrolyte. Table 1 summarises the key parameters and conditions used during the measurements.

### 2.4. Checking the Selectivity of the Electrode

**Figure 5:**
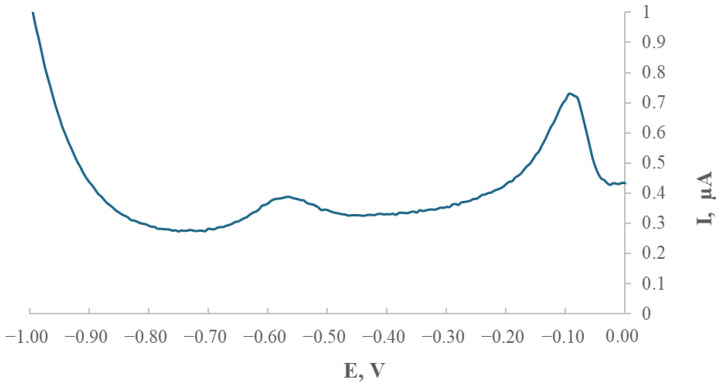
Differential pulse voltammetric voltammogram for the GCE/AgNPs-E1451 electrode recorded in 0.2 M EDTA in the presence of 30 μg/L Tl^+^, 30 μg/L Pb^2+^, 300 μg/L Zn^2+^, 300 μg/L Cd^2+^, 200 μg/L Cu^2+^, deposition time 120 s, deposition potential −1.0 V, step potential 5 mV, differential pulse amplitude 25 mV.

The selectivity of the thallium determination procedure was tested in the presence of four potentially interfering substances contain: Pb^2+^, Zn^2+^, Cd^2+^ and Cu^2+^. Besides these substances tested as interferents were chosen due to their potential occurrence in real samples and their presence did not affect the signals resulting from thallium oxidation. However, in voltammetric determination of thallium, a major challenge lies in the interference caused by cadmium and lead ions, whose reduction potentials are close to that of thallium. This can lead to signal overlap, making it difficult to clearly identify and quantify thallium in the sample. To mitigate this issue, appropriate analytical strategies must be employed, such as the use of complexing agents that limit the influence of cadmium and lead on the thallium signal. One effective approach involves using a 0.2 M EDTA base electrolyte, which, due to its ability to form stable complexes with cadmium and lead ions, significantly reduces their interference in the measurement. The selectivity of the method was tested using the GCE/AgNPs-E1451 electrode in a system containing thallium, lead, zinc, cadmium, and copper ions. The concentration ratio of Tl^+^:Pb^2+^:Zn^2+^:Cd^2+^:Cu^2+^ was set at 3:3:30:30:20. The solution was preconcentrated for 120 s at a potential of −1.0 V, and measurements were taken at the same potential. Figure 5 presents the differential pulse voltammogram recorded for the modified electrode in the presence of these interfering ions.

The peaks observed in the voltammogram were well separated, with the thallium signal appearing at −0.59 V, the zinc signal at a potential of approximately −1.0 V and the copper signal at −0.09 V. The results confirm that thallium can be reliably determined in the presence of other metal ions such as cadmium, lead, copper, and zinc. However, the use of a complexing base electrolyte, such as EDTA, is essential to ensure selective and accurate measurement.

### 2.5. The Application of the Modified Electrode for Thallium Determination in a Soil Sample

To improve sensitivity in thallium detection, the glassy carbon electrode was modified accordingly. This enhancement allows for reliable measurement of trace amounts of thallium, which is particularly important when assessing its presence in environmental matrices such as soil. The modified GCE/AgNPs-E1451 electrode was engineered to offer better selectivity for thallium, helping to reduce the impact of interfering substances commonly found in complex soil samples. As a result, the modification leads to more accurate and consistent analytical results.

**Figure 6:**
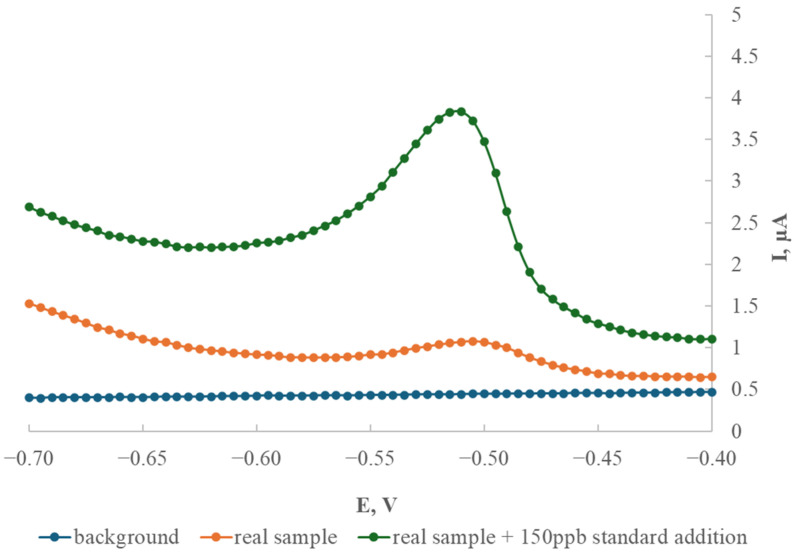
Graph of the relationship between the peak height and thallium concentration for the modified GCE/AgNPs-E1451 electrode recorded in a real sample, deposition time 120 s, deposition potential −1.0 V, step potential 5 mV, differential pulse amplitude 25 mV.

Figure 6 displays a comparison of peak heights obtained from a real soil sample and the same sample spiked with 150 μg/L of thallium standard.

**Figure 7:**
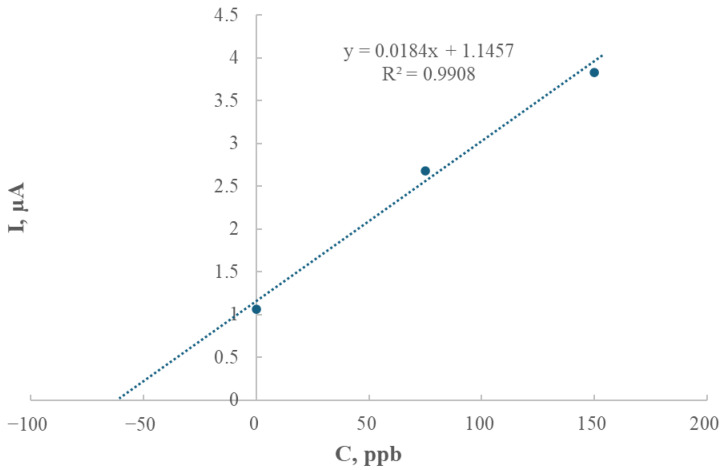
Single standard addition curve (150 μg/L thallium) to a real sample—Bali soil, deposition time 120 s, deposition potential −1.0 V, step potential 5 mV, differential pulse amplitude 25 mV.

Thallium voltammograms were consistent and repeatable when recorded within the narrow potential window of −0.60 V to −0.45 V. Measurements carried out using soil samples from Bali (geographical location: 8°36′37.0″ S 115°15′11.0″ E) proved that thallium can be determined in real environmental samples using the new GCE/AgNPs-E1451 electrode. Bali was selected because the soils on this island are mostly of volcanic origin and are naturally enriched with thallium through volcanic emissions, especially ash. As a result, thallium concentrations in these soils may be elevated, making them particularly relevant for environmental risk assessment and trace element distribution. The standard addition calibration curve is shown in Figure 7.

## 3. Discussion

The development of a glassy carbon electrode (GCE) modified with silver nanostructures stabilised by starch derivatives (GCE/AgNPs-E1451) was aimed at improving thallium detection using voltammetric techniques. A crucial stage of the study involved optimising the deposition potential, which was investigated within the range of −0.8 V to −1.5 V. The most favourable analytical response was observed at −1.0 V, yielding sharp and stable thallium peaks that provided a solid basis for further experiments.

The analysis of peak height as a function of deposition time revealed an almost linear increase (R^2^ = 0.9932) within the range of 40 to 1800 s, indicating that thallium depletion in the solution was negligible under the studied conditions. Subsequent experiments focused on the relationship between thallium concentration and peak current, yielding a calibration curve with excellent linearity (R^2^ = 0.9965), which confirmed the electrode’s suitability for quantitative analysis.

Based on the regression parameters, the limit of detection (LOD) was calculated as 9.21 × 10^−8^ mol/dm^3^ (18.8 μg/L), while the limit of quantification (LOQ) corresponded to 2.76 × 10^−7^ mol/dm^3^ (56.4 μg/L). These values demonstrate the high sensitivity of the modified electrode, making it a reliable tool for detecting trace levels of thallium. The low LOD enables the detection of very small concentrations, whereas the LOQ ensures accurate and repeatable quantification at higher levels.

Such analytical parameters are particularly important in environmental monitoring, where it is essential to detect toxic elements at trace levels. The electrode’s sensitivity allows for effective assessment of thallium content in samples such as water, soil, or other environmental matrices, which is crucial for public health and environmental protection. Additionally, the low detection limits suggest that this electrode can be successfully applied in industrial quality control or scientific research, where precision and sensitivity are essential.

One of the major challenges addressed was the selective detection of thallium in the presence of potential interferents such as cadmium and lead, whose voltammetric signals occur at similar potentials. The use of 0.2 M EDTA as the base electrolyte enabled effective separation of peaks in the voltammograms, thus minimising signal overlap and improving identification accuracy. To further validate the electrode’s practical applicability, an analysis of a soil sample from Bali was performed, confirming its effectiveness not only under laboratory conditions but also in real sample matrices.

Regular cleaning of the working electrode and repeating the surface modification every 10–12 measurements proved essential for maintaining the electrode’s stability and precision, ensuring consistently well-defined thallium peaks. The application of the GCE/AgNPs-E1451 modified electrode significantly enhances the performance of voltammetric thallium determination. A deposition potential of −1.0 V was identified as optimal, providing reliable and reproducible analytical results. The electrode demonstrated a broad applicability range, enabling accurate quantitative analysis of thallium in various matrices.

In comparison to conventional mercury-based electrodes, the modified GCE/AgNPs-E1451 is more environmentally friendly and safer to handle, making it a viable alternative for both laboratory and field applications. The effectiveness of this electrode in real sample analysis was confirmed by applying the method to a soil sample from Bali. The thallium content in this sample, determined by extrapolation from the standard addition curve, was found to be 1.55 mg/kg (equivalent to 62.26 μg/L).

When compared with literature-reported thallium levels in soil, which typically range from 0.1 to 2.0 mg/kg [18], the concentration obtained in this study indicates a relatively elevated thallium content—higher than those reported for Canadian soils, where average concentrations in former urban and rural parklands in Ontario reached 0.79 mg/kg. For Scottish soils, mean thallium values ranged from 0.1 to 0.8 mg/kg, while in French soils an average concentration of 1.51 mg/kg was reported [19].

Investigations of elevated thallium concentrations in soils from the Lanmuchang region in southwestern Guizhou, China, have been linked to natural sulfide mineralisation processes, particularly involving Tl-rich ore deposits. The results of the present study, therefore, align with the broader context of environmental thallium distribution and support the application of the modified GCE/AgNPs-E1451 electrode as a robust analytical tool for monitoring this toxic element in complex environmental samples.

Thallium concentrations in soils from mining areas have been reported to range from 40 to 124 mg/kg, while materials eroded from slopes contained 20–28 mg/kg, alluvial sediments downstream from 14 to 62 mg/kg, and undisturbed natural soils from 1.5 to 6.9 mg/kg. These values indicate that both natural erosion of thallium-mineralised soils and mining activities are responsible for the spread of elevated thallium concentrations in the environment [20].

The LOD obtained in this study was compared to values reported in earlier works. In reference [21], describing a similar electrode modification (GCE/Cu-MOF), the linearity range was 0.5–700 μg/L, with a detection limit (LOD) of 0.11 μg/L. A significantly lower LOD of 0.86 ng/mL was reported in [22], with a linearity range from 3.0 to 250 ng/mL. This sensor characterised good repeatability and was applied to the determination of thallium (Tl^+^) in water, hair samples and certified reference materials.

The results obtained from the Indonesian soil sample enabled a comparative analysis with other heavy metals. The lead (Pb) content ranged from 0.01 to 142.02 mg/kg, exceeding the quality standard of 100 mg/kg [23]. The mean values obtained were 19.59 mg/kg for soil at a depth of 10–20 cm (top layer), 20.13 mg/kg for soil at a depth of 50–60 cm (middle layer) and 13.76 mg/kg for soil at a depth of 90–100 cm (bottom layer). The Cd content ranged from 0.15 to 18.1 mg/kg, exceeding the quality standard of 1.5 mg/kg [23]. The average values obtained were 6.44 mg/kg for soil at a depth of 10–20 cm, 7.19 mg/kg for soil at a depth of 50–60 cm, and 6.65 mg/kg for soil at a depth of 90–100 cm. Both exceed the typical threshold values specified in the Polish Regulation of the Minister of the Environment of 9 September 2002 on soil and land quality standards [24].

A separate study using a reduced graphene oxide-modified electrode for monitoring thallium in grain-based products demonstrated a linear anodic stripping current response for thallium concentrations ranging from 9.78 × 10^−9^ to 97.8 × 10^−9^ mol/dm^3^, with a calculated LOD of 1.229 μg/L (6.01 × 10^−9^ mol/dm^3^). The method was successfully applied to real food samples [24].

The GCE/AgNPs-E1451 electrode presented in this study demonstrates significantly higher sensitivity than the GCE/AuNPs–LS/Hg electrode, which reported a detection limit of 1.4 × 10^−7^ mol/dm^3^ [25]. Moreover, the elimination of toxic mercury renders the GCE/AgNPs system a more environmentally sustainable alternative.

## 4. Materials and Methods

### 4.1. Synthesis and Characteristics of Silver Nanoparticles

The composite material investigated in this study is based on silver nanostructures stabilised with a hydrolysate derived from a doubly modified starch—acetylated and oxidised—commercially available as E1451 and sourced from WPPZ (Strzałkowo, Poland). Acetylated oxidised starch is obtained through a two-step modification process: native potato starch is first oxidised using sodium hypochlorite and then acetylated with acetic anhydride. This dual modification significantly enhances the functional properties of the starch compared to its native form. When dissolved at elevated temperatures, the modified starch forms a transparent, highly stable gel, making it well-suited for use as a stabilising agent in nanomaterial synthesis.

Nanosilver was synthesised starting from an ammoniacal complex of silver, [Ag(NH_3_)_2_]^+^, prepared to contain silver ions at a concentration of 1 g/dm^3^. The reagents—silver nitrate and aqueous ammonia—were obtained from POCh (Gliwice, Poland). To the reaction system, a starch-based modifier was introduced at 10 g/dm^3^. The synthesis was carried out at a constant temperature of 80 °C, with the mixture continuously stirred using a magnetic stirrer.

The heating phase lasted one hour, after which the external heat source was removed. However, the system remained under continuous stirring for a period of seven days to allow the reaction to proceed further under ambient conditions.

Characterisation of the resulting silver nanostructures involved UV-vis spectrophotometry using an OceanOptics USB4000 (Orlando, FL, USA) instrument to monitor optical properties. Particle size distribution was analysed with a Zetasizer Nano ZS (Malvern Instruments, Malvern, UK), which applied a non-invasive backscattering technique. The same instrument, equipped with an automatic titration unit, was used to assess electrophoretic mobility based on the Doppler phenomenon.

**Figure 8:**
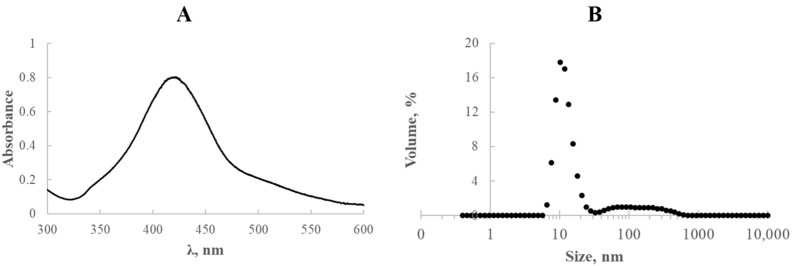
Representative physicochemical properties of AgNPs stabilized with E1451: (**A**) UV-vis spectroscopic profile; (**B**) size distribution of nanoparticles.

The essential physicochemical features of the fabricated silver nanostructures are depicted in Figure 8.

The synthesised solutions exhibited a well-defined absorption band centred around 420 nm, characteristic of plasmonic silver nanoparticles (Figure 8A). The particle size distribution of the resulting AgNPs is shown in Figure 8B. As illustrated in the inset, particle diameters ranged from 6 to 615 nm, with the dominant fraction corresponding to nanoparticles approximately 10.1 nm in diameter (17.8%).

Given that the zeta potential provides valuable insight into the stability of colloidal dispersions, this parameter was also examined for the silver nanostructures obtained. According to literature data, systems with an absolute zeta potential value exceeding ±30 mV are generally considered electrostatically stable, as sufficient repulsive forces between particles help prevent aggregation. Values between ±10 mV and ±30 mV indicate moderate (or intermediate) stability, while absolute values below ±10 mV typically suggest poor colloidal stability and a high tendency toward agglomeration [26,27].

In the case of the synthesised silver nanostructures, the measured zeta potential was −20 mV, classifying the system as moderately stable. Although this value falls short of the threshold for strong electrostatic stabilisation, the nanoparticles demonstrated sufficient stability for use in electrode applications. Specifically, when applied as modifiers for glassy carbon electrodes (GCE), no noticeable issues related to aggregation were observed during electrode preparation.

### 4.2. Mineralisation of the Soil Samples

Soil samples from Bali were mineralised in an open system using a graphite furnace, following the procedure previously described by Karbowska et al. [25].

### 4.3. Preparation and Testing of a Silver Nanostructure-Modified Electrode Stabilised with Starch Derivatives

A freshly cleaned glassy carbon electrode with a diameter of 3 mm was modified by applying 4 µL of a silver nanostructure suspension stabilised with a doubly modified starch hydrolysate. Once the material was drop-cast onto the surface, the electrode was left to dry and then integrated into the measurement system, which consisted of a saturated calomel electrode (Hg_2_Cl_2_|Cl^−^ in 3 M KCl) as the reference electrode and a platinum wire as the counter electrode.

Measurements were carried out using differential pulse anodic stripping voltammetry (DP-ASV) in a 0.2 M EDTA solution at pH 4.5. Before each experiment, the electrolyte was deoxygenated by purging with nitrogen. The procedure was conducted under strictly controlled conditions: the preconcentration time was set to 120 s, with a preconcentration potential of −1.0 V. The pulse amplitude was 25 mV, and the step potential was 5 mV. The scan rate used during the voltammetric sweep was 0.01 V/s. Additionally, each measurement included a pulse time of 0.07 s and an equilibration period of 10 s before data acquisition began.

### 4.4. Preparation of the GCE/AgNPs-E1451 Electrode for Analytical Measurements

Before starting any measurements, the electrode must undergo a series of steps to ensure its surface is properly prepared. This process improves the quality and repeatability of the results.

- Initial cleaning with alumina powder

The electrode is first polished using fine alumina. This step removes impurities and refreshes the surface, making it suitable for further treatment.

- Washing off polishing residues

To get rid of alumina particles left after polishing, the electrode is placed in an ultrasonic bath. This ensures that no solid residues remain on the surface.

- Applying a surface modifier

After cleaning, a modifying agent is carefully applied. This substance adjusts the surface properties of the electrode, allowing it to interact selectively with the target analytes.

- Drying phase

The modified electrode is dried at a temperature of 80 °C. This helps the modifier to bind firmly to the surface and stabilises the layer.

- Ready for testing

Once dry, the electrode is ready to be used in measurements. At this point, all preparatory steps are complete.

### 4.5. Morphological Characterisation of the Modified Electrode Using SEM

**Figure 9:**
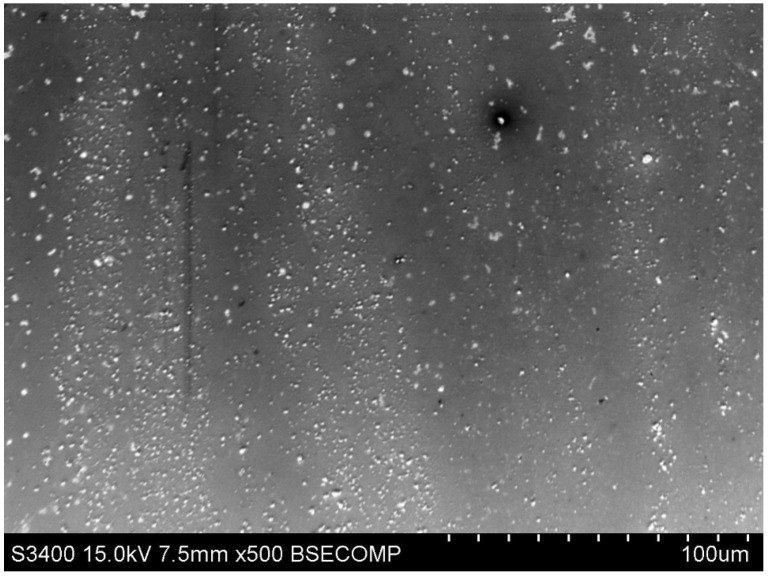
Surface of the GCE/AgNPs-E1451 Electrode—SEM Image.

The surface morphology of the modified GCE/AgNPs-E1451 electrode was examined using scanning electron microscopy (SEM). As shown in Figure 9, the SEM image reveals a distinctly porous structure accompanied by visible surface folding. These morphological features significantly enhance the sorption capacity of the electrode, which is beneficial for analytical applications requiring increased surface interaction.

### 4.6. Determination of Thallium Ions Using Differential Pulse Anodic Stripping Voltammetry

To verify and confirm the sensitivity of the modified GCE/AgNPs-E1451 electrode toward thallium ions, as well as to stabilise the measurement system and characterise the modified electrode, a series of experiments was conducted using differential pulse anodic stripping voltammetry (DPASV).

During the study, the experimental conditions for thallium determination were optimised. Each sample was subjected to a 10-min deoxygenation step by purging with purified nitrogen gas. Thallium quantification was carried out using the standard addition method. After each addition, a voltammetric response was recorded in the form of a stripping curve.

## 5. Conclusions

The results obtained in this study contribute significantly to the advancement of thallium detection technology using glassy carbon electrodes modified in voltammetric techniques. The identified accumulation potential of −1.0 V proved to be a crucial factor for achieving optimal analytical outcomes, allowing for clear and distinct thallium peaks that enhance both the precision and sensitivity of the electrode. Investigations into the relationship between peak current and thallium concentration, based on the calibration curve, demonstrated high electrode sensitivity, highlighting its promise as a tool for accurate quantitative analysis of thallium.

Experiments designed to rule out thallium depletion in the solution, along with the analysis of peak height dependence on accumulation time, confirmed the electrode’s stability and durability—key attributes for practical applications. Selectivity tests further validated the electrode’s ability to detect thallium even in the presence of interfering ions such as cadmium and lead, showcasing its potential in challenging environments where electrochemical similarities might complicate measurements.

The application of the modified electrode for determining thallium in soil samples confirmed its effectiveness under real-world conditions. However, the need for regular cleaning and repeated modification every ten to twelve measurements, especially during extended measurement sessions, represents a limitation that calls for further research focused on optimising the longevity of this modification.

Overall, the developed modification offers a highly effective tool within electrochemistry, particularly for precise quantitative analyses of thallium across various matrices. The findings lay a solid foundation for ongoing efforts to improve and refine this technology.

## Figures and Tables

**Table 1:** Measurement parameters used in DP-ASV analysis.

Parameter	Value
Deposition time	120 s
Deposition potential	−1.0 V
Differential pulse amplitude	25 mV
Step potential	5 mV
Starting potential	−0.8 V
Ending potential	−0.4 V
Scan rate	0.01 V/s
Differential pulse duration	0.07 s
Solution equilibration time	10 s

## Data Availability

Data are obtainable from the corresponding authors upon reasonable request.
